# The Contribution of Sound Intensity in Vocal Emotion Perception: Behavioral and Electrophysiological Evidence

**DOI:** 10.1371/journal.pone.0030278

**Published:** 2012-01-23

**Authors:** Xuhai Chen, Jianfeng Yang, Shuzhen Gan, Yufang Yang

**Affiliations:** 1 State Key Laboratory of Brain and Cognitive Science, Institute of Psychology, Chinese Academy of Sciences, Beijing, China; 2 Graduate University of Chinese Academy of Sciences, Beijing, China; University of Chicago, United States of America

## Abstract

Although its role is frequently stressed in acoustic profile for vocal emotion, sound intensity is frequently regarded as a control parameter in neurocognitive studies of vocal emotion, leaving its role and neural underpinnings unclear. To investigate these issues, we asked participants to rate the angry level of neutral and angry prosodies before and after sound intensity modification in Experiment 1, and recorded electroencephalogram (EEG) for mismatching emotional prosodies with and without sound intensity modification and for matching emotional prosodies while participants performed emotional feature or sound intensity congruity judgment in Experiment 2. It was found that sound intensity modification had significant effect on the rating of angry level for angry prosodies, but not for neutral ones. Moreover, mismatching emotional prosodies, relative to matching ones, induced enhanced N2/P3 complex and theta band synchronization irrespective of sound intensity modification and task demands. However, mismatching emotional prosodies with reduced sound intensity showed prolonged peak latency and decreased amplitude in N2/P3 complex and smaller theta band synchronization. These findings suggest that though it cannot categorically affect emotionality conveyed in emotional prosodies, sound intensity contributes to emotional significance quantitatively, implying that sound intensity should not simply be taken as a control parameter and its unique role needs to be specified in vocal emotion studies.

## Introduction

Humans communicate emotion vocally by modulating acoustic cues such as pitch, intensity, rhythm, and vocal qualities, termed as emotional prosody or vocal emotion. Banse and Scherer [1] suggested that each emotion has its unique physiological “imprint” and is expressed in a unique manner. For instance, compared with utterance with no emotional expression, anger is characterized by fast speech rate, high mean fundamental frequency (F0), F0 variability and sound intensity. Relative to numerous studies addressing the role of pitch [2]–[4], speech rate [5], and voice quality [6]–[7], the contribution of sound intensity as well as its neural correlates in vocal emotion perception, to our knowledge, remains largely ignored. Therefore, the current study aims to address the role of sound intensity and its neural underpinnings in vocal emotion perception.

Numerous studies indicated that sound intensity is one of the most important acoustic parameters to convey emotion. In a review analyzing the vocal indicators of various emotions, Pittam and Scherer [8] stated that energy is one of the three perceptual dimensions (energy, pitch, and time) on which most vocal cue based. These results were confirmed by their following study [1], suggesting that “intense” emotions like hot anger and panic fear showed higher mean energies than those not so intense like sad and shame. Similarly, Juslin and Laukka [9] tested how intended emotion intensity influenced acoustic cues and found that portrayals of the same emotion with different intensity yielded different patterns of acoustic cues, including higher voice intensity for the strong emotions than the weak emotions. Moreover, it is claimed that the function of sound intensity is pretty important, as indicated by the fact that people most often report voice cues such as loudness or talking speed to judge the emotional states of others in everyday life [10].

Indeed, sound intensity is one of the most elementary features of auditory signals, and change in the intensity of vocal sounds might be emotionally relevant. In a study with oddball paradigm, it was found that intensity change elicited a mismatch negativity (MMN) and P300 effect with different patterns for vocal and non-vocal materials, suggesting that simple acoustic change recruits more processing resources if it is socially relevant [11]. Additionally, human subjects overestimate the change of rising intensity sounds compared with falling intensity sounds, as recent studies indicated that rising sound intensity is an elementary warning cue eliciting adaptive and emotional responses by recruiting attentional and physiological resources [12]–[14]. Moreover, an imaging study suggested that discrimination of sound intensity involves two different cortical networks: a supramodal right frontoparietal network responsible for allocation of sensory attentional resources, and a region of secondary auditory cortex specifically involved in sensory computation of sound intensity differences [15].

Distinct from aforementioned line of researches emphasizing the contribution of sound intensity or energy in vocal emotion communication, some other researches claimed that sound intensity contours do not bring any significant information when used alone [16]. In line with this opinion, most studies investigating the neural correlates underlying perception of vocal emotion employed stimuli which were matched for acoustic energy parameters [17]–[20]. Obviously, such approaches are limited by the fact that emotional information in the voice is transmitted via certain acoustic features [1] and leave the specific role of sound intensity in vocal emotion perception unknown as yet. Thus, there remains controversy regarding the role of sound intensity in vocal emotion, which is likely due to the fact that vocal expression can be described in terms of discrete emotion or dimensional construct. Although most studies of vocal expression focused on discrete emotion, it has been suggested that the affective state expressed often in spontaneous speech are not best characterized as full-blown emotion episodes like basic emotion, but rather as milder forms of emotion states [21]. Sound intensity might be trifle when we describe vocal emotion in terms of discrete emotion [16], but it might be very important for some dimensions when vocal emotion is regarded as a dimensional construct [1], [9]. Therefore, we hypothesized that sound intensity has a unique role in vocal emotion communication, that is, the change of sound intensity could affect the emotional significance of vocal emotion although it might not categorically change its emotionality.

To test this hypothesis, two experiments were carried out. Firstly, to test whether sound intensity alone can bring any significant variation in emotion perception, participants were asked to rate the anger level for angry and neutral prosodies before and after their mean sound intensities were modified. And then, electroencephalogram (EEG) were recorded for matching and mismatching sentence prosodies created through cross-splicing method [22] while participants performing emotional feature or sound intensity congruousness judgment. In this paradigm, mismatching prosodies deviated from the preceding contexts and thus led to expectation violation. In comparison with matching prosodies, deviations in mismatching prosodies were reported to elicit N2-P3 complex enhancement in active condition, indicating a process of deviation detection and integration [23]. In line with previous studies [20], [23], we hypothesized that all mismatching prosodies would elicit N2-P3 complex in comparison to matching prosodies, but its latency and amplitude would be modulated by sound intensity modification and task demands if sound intensity has a quantitative effect on vocal emotion expression.

Besides phase-locked ERPs, neural oscillations, which reflect neural rhythm changes of ongoing EEG time-locked to stimulus onset, may provide more information about the contribution of sound intensity in vocal emotion communication. Both external stimuli and internal mental events can induce event-related synchronization/desynchronization that are identified by the increase/decrease of spectral power at specific frequency band [24]. The current study focused on the low frequency oscillation given that previous studies suggested that these band activities contribute to memory encoding [25] and are associated with emotional discrimination [26]–[30]. Moreover, it has been documented that expectation violation or rule violation are related to a relative increase in power of theta band [31]–[36] and theta band activities have been shown to underlie P300 ERP activity [37]. Furthermore, the studies by Tzur and Berger [33], [34] showed that theta activity is sensitive to the salience of the violation, that is, the degree of deviation of the conflicting stimulus from the expected one. Based on these findings, the current work expected that the deviation in emotional prosodies would induce theta band synchronization and their power would be modulated by sound intensity modification.

## Results

### Experiment 1

#### Behavioral Results

**Figure 1:**
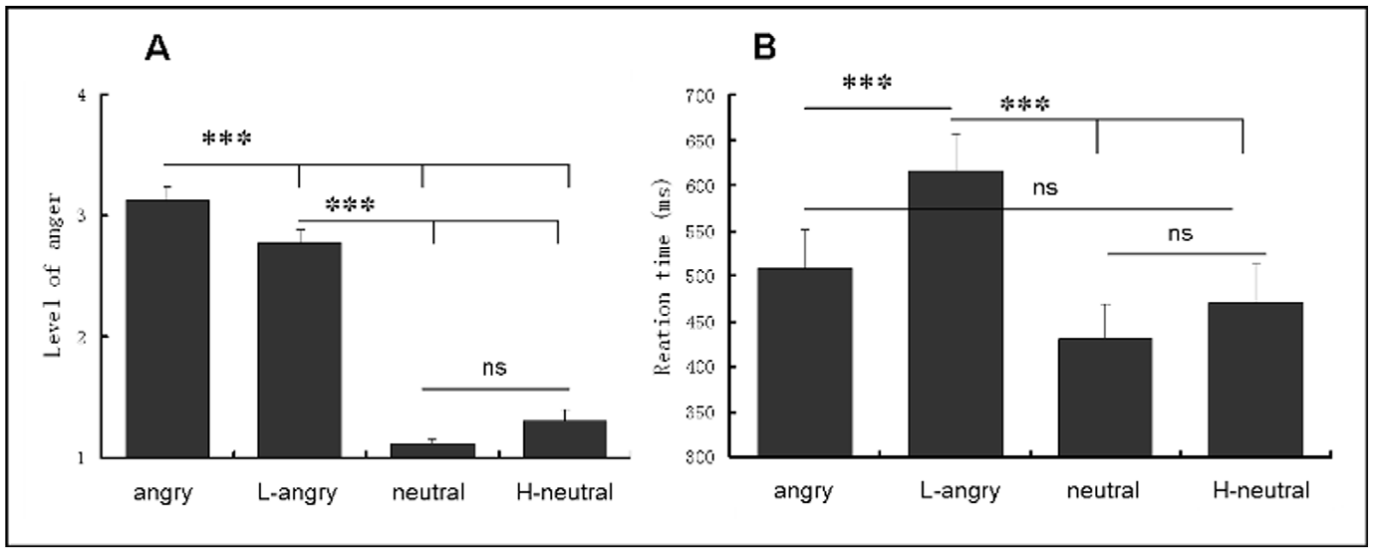
Rating scores (A) and reaction times (B) for four types of prosodies in Experiment 1 (M±se). In this figure, as in the following ones, Asterisk (*) indicates a significant response difference at P<0.05, ** at P<0.01, and ** * at P<0.001.

The mean rate of anger level and the reaction times for four types of materials were shown in Figure 1. The anger level for angry prosodies was the highest, followed by L-angry, H-neutral, and neutral prosodies. The ANOVA on anger level yielded a significant main effect of Prosody-type [*F*(3,51) = 316.24, *p*<.001,*η* ^2^ = .95]. Pairwise comparison revealed that the anger level for angry prosodies was higher than those for all other three types of prosodies (*ps*<.001), and the L-angry prosodies sound angrier than H-neutral and neutral prosodies (*ps*<.001), while no significant difference between H-neutral and neutral was found (*p*>.1). The ANOVA on the reaction times yielded significant main effect of Prosody-type [*F*(3,51) = 12.31, *p*<.001,*η* ^2^ = .42]. Following pairwise comparison found that the reaction times for L-angry prosodies were significantly longer than those for the other three types of prosodies (*ps*<.001), while no salient difference was found between the other three types of prosodies.

Distinct from acoustically analyzing the contribution of various parameters in previous studies [1], [8], this experiment manipulated the mean sound intensity, and thus the difference in anger rating could be merely attributed to the change of sound intensity. Consistent with the claim that changing intensity contours alone cannot bring any significant information [16], we observed that sound intensity raising could not significantly enhance the anger level of neutral prosodies and reduced sound intensity angry prosodies still sound angry. However, decreasing sound intensity conspicuously weaken the angry level and prolonged the reaction times for angry prosodies, suggesting that sound intensity do have a role in vocal emotion encoding [1], [9], at least in expression of anger.

Despite the fact that the current results provided clear evidence for the role of sound intensity in vocal emotion perception, the quantitative variation in angry level rating might not completely rule out the possibility that changing sound intensity convey another emotion other than angry although no salient angriness change happened. Thus, we conducted Experiment 2 using context violation paradigm which could direct participants' attention to the emotional categorical change directly. Moreover, the brain responses were recorded to test whether electrophysiological data could provide consistent evidence.

### Experiment 2

#### Behavioral Results

**Figure 2:**
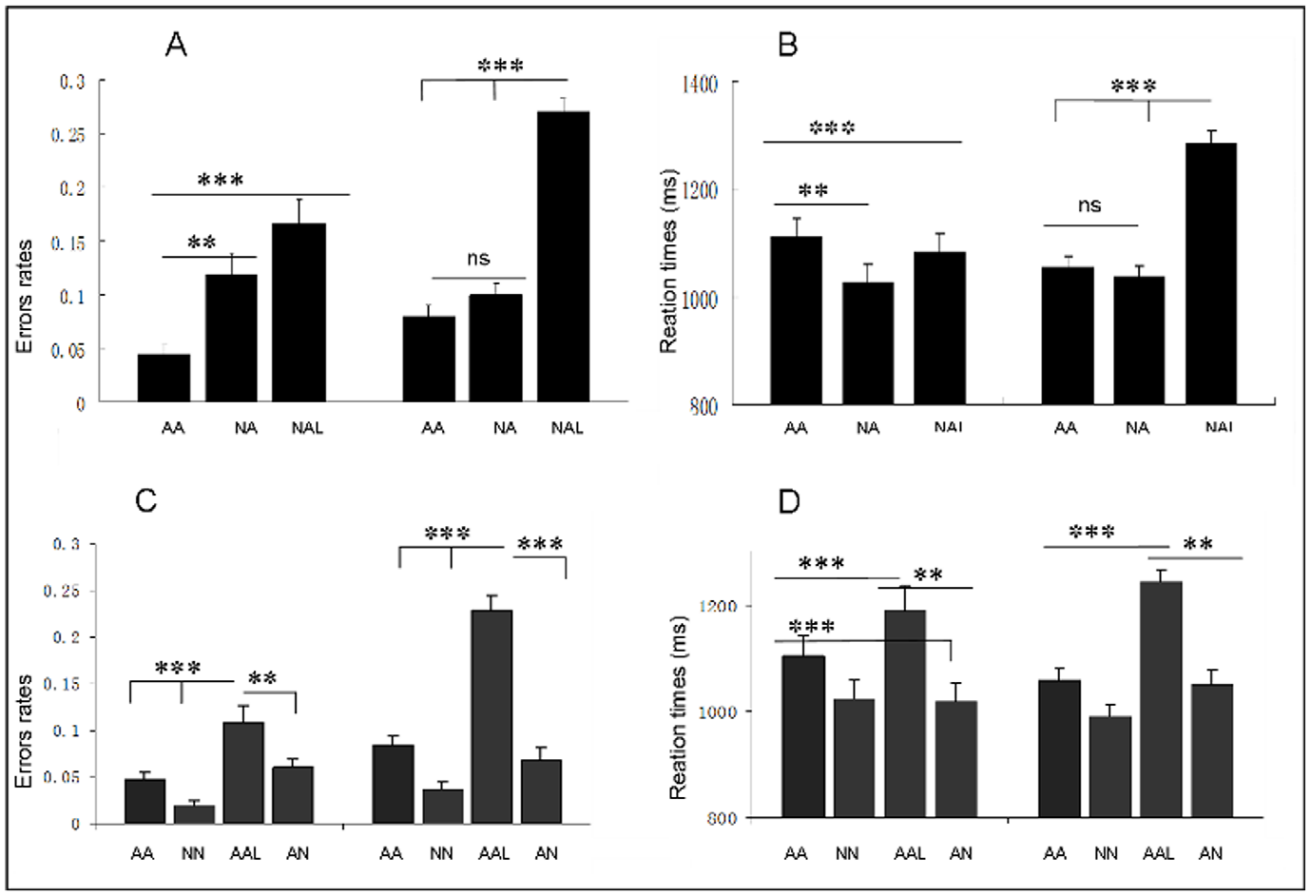
Behavioral results of Experiment 2. Error rates (A, M±se) and Reaction times (B) for three types of critical prosodies under two task conditions (left: emotion task; right: intensity task). Error rates (C) and Reaction times (D) for fillers and “AA” baseline prosodies under two task conditions.

Error rates and RTs were calculated for each participant and corrected by 2.5 SD of the mean, as shown in Figure 2. And then two sets of data were calculated in separate repeated measures ANOVAs with *Task* (emotion judgment vs. intensity judgment), and *Prosody-type* (“AA”, “NA” and “NAL”) as within subject factors. The result revealed that error rates were higher in the intensity than emotional condition [*F*(1,14) = 6.55, *p*<.05, *η* ^2^ = .32], and different between three prosodic types [*F*(2,28) = 61.82, *p*<.0001,*η* ^2^ = .82]. Moreover, two-way interaction of *Task*×*Prosodic-type* was significant [*F*(2,28) = 14.41, *p*<.001, *η* ^2^ = .51]. Further simple effect analyses found that the error rates were higher for “NAL” and “NA” than that for “AA” prosodies in emotion task condition (*p*<.001 and *p*<.01 respectively), whereas no salient difference between the former two were observed. However, in intensity task condition, the error rates for “NAL” were significantly higher than those for “NA” and “AA” prosodies (*p*<.001), while there was no conspicuously difference between the later two types of prosodies. The ANOVA on RT yielded significant main effect of *Prosodic-type* [*F*(2,28) = 29.26, *p*<.001, *η* ^2^ = .68] and two-way interaction of *Task*×*Prosodic-type* [*F*(2,28) = 36.17, *p*<.001, *η* ^2^ = .72]. Further simple effect analyses showed that the RTs were slower for “AA” than for “NA” in emotion task condition (*p*<.05), and in intensity task condition, the RTs for “NAL” prosodies were significantly slower than those for “NA” and “AA” prosodies (*ps*<.001), while there was no salient difference between the later two types of prosodies.

The Error rates and RTs for the fillers were compared with the behavioral response for “AA” prosodies (see Figure 2). The result for error rates showed a significant main effect of *Prosodic-type* [*F*(3,42) = 44.25, *p*<.001, *η* ^2^ = .76] and two-way interaction of *Task*×*Prosodic-type* [*F*(3,42) = 10.99, *p*<.001, *η* ^2^ = .44]. Further simple effect analyses showed that the Error rates were higher for “AAL” than for other kinds of prosodies (*ps*<.001 or *ps*<.01) in both task conditions, while no other difference reach the significance level. Moreover, The result for RTs showed a significant main effect of *Prosodic-type* [*F*(3,42) = 72.92, *p*<.001, *η* ^2^ = .83] and two-way interaction of *Task*×*Prosodic-type* [*F*(3,42) = 4.40, *p*<.05, *η* ^2^ = .19]. Further simple effect analyses showed that the RTs were longer for “AAL” than for other kinds of prosodies (*p*<.001 or *p*<.01) in both task conditions, while the RTs were longer for “AA” than for “NN” and “AN” (*p*<.001) in emotion task and longer for “AA” than for “NN” prosodies (*p*<.01) in intensity condition. No other difference reached the statistical significance level (*p*>.1).

### Electrophysiological Results

#### Raw ERP analysis

**Figure 3:**
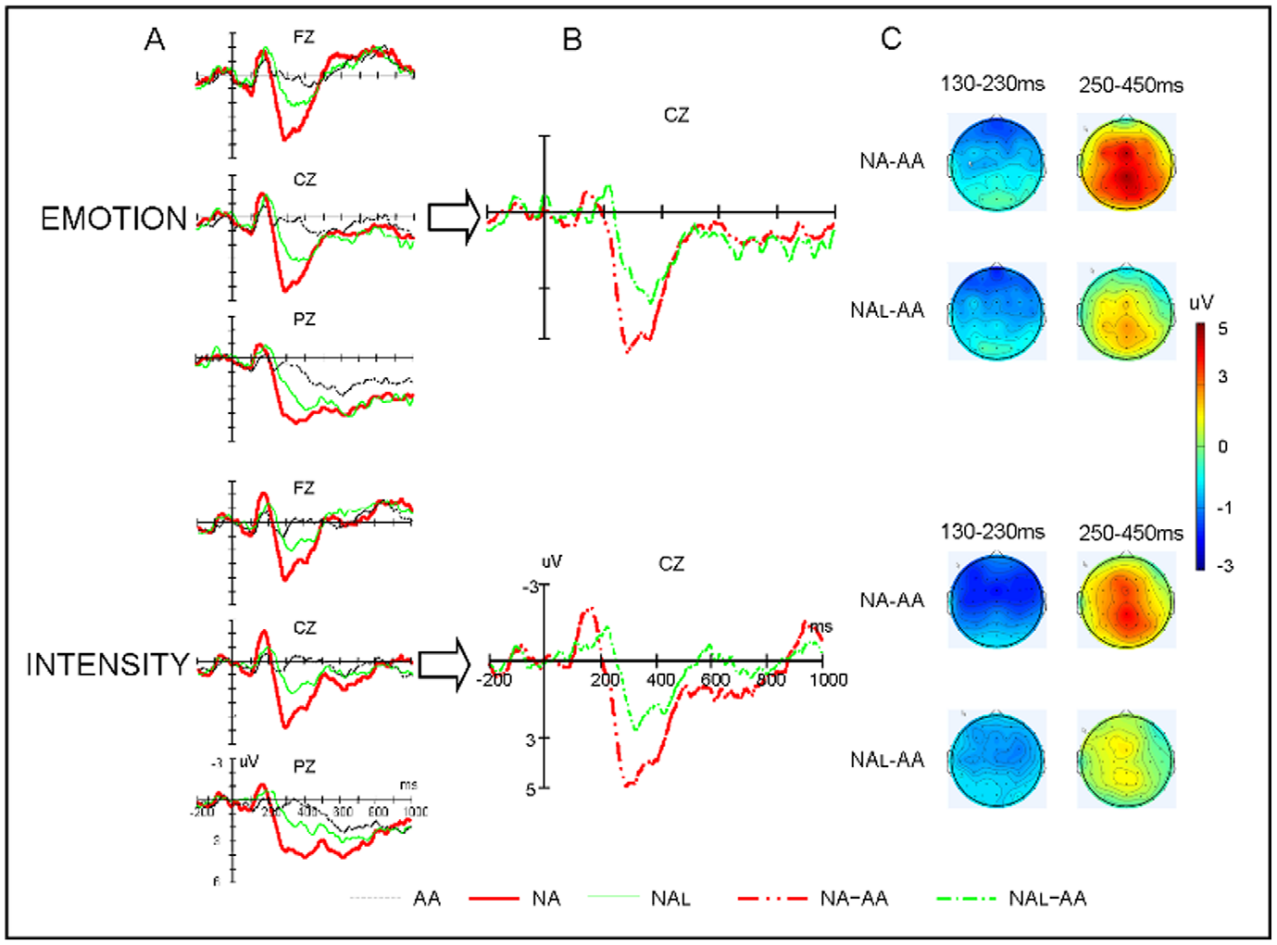
Grand-average ERP waveforms for critical prosodies. A: ERPs elicited by three types of prosodies at selected electrode sites. In this figure, the amplitude (in microvolt) is plotted on ordinate (negative up) and the time (in milliseconds) is on abscissa. B: Difference waves (NA minus AA versus NAL minus AA). C: Topographies of difference curves (viewed from the top) in the selected time periods.

The repeated measures ANOVAs at 130–230 msec interval showed a significant main effect of *Task* [*F*(1,14) = 5.05, *p*<.05,*η* ^2^ = .27], with the ERPs more negative going for intensity task than for emotion task. Also significant were the main effect of *Prosody-type*, [*F*(2,28) = 9.81, *p*<.001, *η* ^2^ = .41], and interaction of *Prosody-type*×*Laterality*, [*F*(4,56) = 2.89, *p*<.05, *η* ^2^ = .17], see Figure 3 for a graphic illustration. Further simple tests showed that in both task conditions “NA” prosodies elicited more negative going deflection in comparison with “AA” prosodies over all hemispheres (*p*<.001), whereas “NAL” prosodies only elicited more negative going ERPs than the “AA” prosodies did over right hemisphere (*p*<.01). Moreover, the differences of ERPs elicited by “NAL” and “NA” prosodies were marginally significant over left hemisphere (*p* = .068).

The ANOVA on mean amplitude in the 250–450 msec time window revealed a significant main effect of *Task* [*F*(1,14) = 4.61, *p*<.05, *η* ^2^ = .25], with the ERPs more positive going for emotion task than for intensity task. Also significant were the main effect of *Prosody-type* [*F*(2,28) = 28.37, *P*<.001, *η* ^2^ = .67], two-way interaction of *Prosody-type*×*Laterality* [*F*(4,56) = 17.36, *p*<.05, *η* ^2^ = .55], three-way interaction of *Task*×*Laterality*×*Prosody-type*, [*F*(4,56) = 4.36, *p*<.05, *η* ^2^ = .24], and three-way interaction of *Laterality*×*Prosody-type*×*Sagittality* [*F*(8,112) = 6.40, *p*<.001, *η* ^2^ = .31]. Following simple effect tests showed that the “NA” and “NAL” prosodies elicited more positive going deflection compared to “AA” prosodies over all regions under both task conditions (*ps*<.001 and *ps*<.01 respectively). Moreover, the positivities elicited by “NA” were more positive going than those elicited by “NAL” prosodies over all regions, (*ps*<.001 or *ps*<.05). In addition, the simple effect analysis forthe interaction of *Laterality*×*Prosody-type*×*Sagittality* revealed that “NA” prosodies elicited more positive going deflection compared to “AA” and “NAL” prosodies over all regions and hemispheres (*ps*<.001and *ps*<.01 respectively), whereas “NAL” prosodies only elicited more positive going deflection than “AA” did over central-middle, central-left, and anterior-middle areas (*ps*<.01).

#### Difference wave analysis

The repeated measures ANOVA showed that the N2 amplitudes had no significant effects involving *Prosody-type* (*p*s>.1). However, the N2 peak latencies were shorter for “NA-AA” than “NAL-AA”, [*F*(1,14) = 14.68, *p*<.01, *η* ^2^ = .51], and is also shorter in intensity task than in emotion task, [*F*(1,14) = 14.67, *p*<.01, *η* ^2^ = .51]. Additionally, there was a significant interaction of *Electrode*×*Prosody-type*×*Task*, [*F*(17,238) = 2.94, *p*<.05, *η* ^2^ = .17]. Simple analysis found that latencies were shorter for “NA-AA” than “NAL-AA” at F3, FC3, C3, CP3, CPZ, and PZ (*ps*<.05) under emotion task condition, while the difference in intensity task was only significant at C4, P4, and PO4 (*ps*<.05). The analysis of P3 showed larger amplitudes for “NA-AA” than “NAL-AA”, [*F*(1,14) = 7.84, *p*<.05, *η* ^2^ = .36]. The two way interaction of *Electrode*×*Prosody-type* was significant, [*F*(17,238) = 2.71, *p*<.05, *η* ^2^ = .16], and following simple analysis found that P3 had larger amplitudes for “NA-AA” than “NAL-AA” at F3, FZ, F4, FCZ, FC4, CZ, CZ, C4, CPZ, CP4, PZ and P4 (*p*s<.05). Moreover, “NA-AA” evoked shorter P3 peak latencies than “NAL-AA” did [*F*(1,14) = 14.04, *p*<.01, *η* ^2^ = .50] and were longer in intensity task than in emotion task, [*F*(1,14) = 7.83, *p*<.05, *η* ^2^ = .36]. Therefore, our additional analysis of difference waves confirmed the results shown by the raw ERP analysis, indicating reduced processing of emotional prosody change when sound intensity decreased.

#### ERO analysis

**Figure 4:**
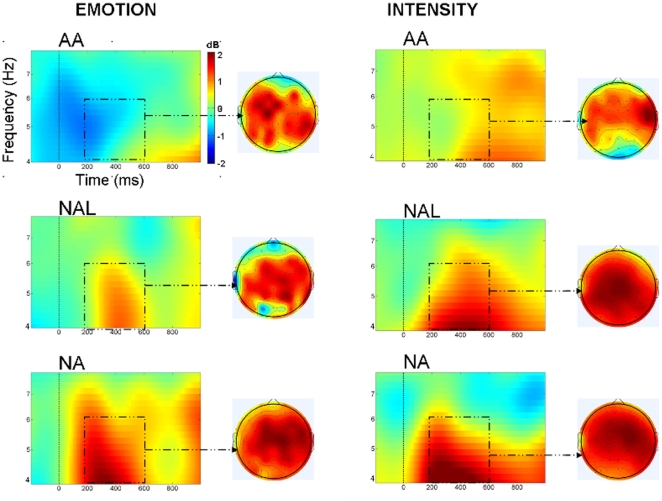
The average oscillatory activities for various critical prosodies and task conditions. The time–frequency map shows oscillatory activities at Cz electrodes the over time (x-axis; 0 is onset of splicing point) and frequency (y-axis). Red colors indicate more power increase and blue colors indicate more power decrease relative to baseline. Topographical map show data taken from a 100- to 600-ms, 4- to 6-Hz window.

Figure 4 displayed the spectrograms and topographical maps for various types of prosodies under two task conditions. As shown in these figures, all types of prosodies induced theta band synchronization over frontal-central areas, which was confirmed by significant main effect of *Sagittality* [*F*(2,28) = 7.24, *p*<.01,*η* ^2^ = .34], with the power centrally peaking. More importantly, while the matching prosodies induced somewhat small theta band synchronization, the mismatching prosodies induced strong power increasing regardless of task demands. The repeated measures ANOVAs confirmed these results by showing a significant main effect of *Prosody-type* [*F*(2,28) = 16.92, *p*<.001,*η* ^2^ = .55]. Post hoc comparison revealed that theta band power was larger for “NA” and “NAL” prosodies (*ps*<.05) in comparison with that induced by AA prosodies. Moreover, the theta band power for “NA” was also larger than that for “NAL” (*p*<.05). Although the power under sound intensity conditions looks larger than that under emotion condition, the statistical analysis found no significant difference. In addition, no significant interaction involving prosodic type and task was found.

#### Analysis for Fillers (see Figure 5)

**Figure 5:**
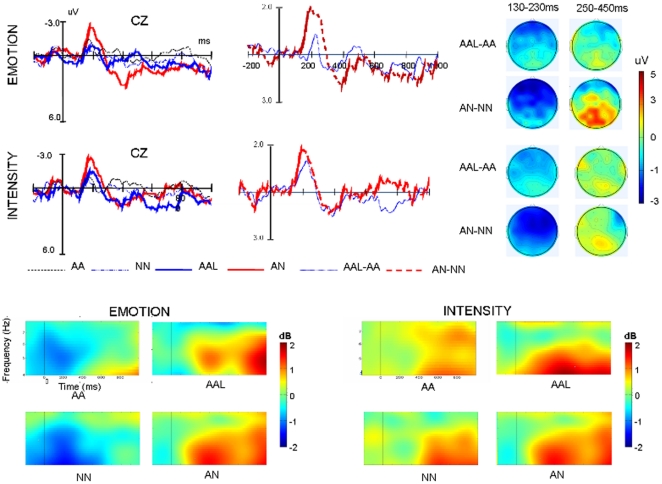
Grand-average ERP waveforms and average oscillatory activities for for fillers and “AA” baseline prosodies under two task conditions. (Up): ERPs elicited by fillers and “AA” prosodies and difference waves at CZ and Topographies of difference curves. (Below): The time-frequency map shows oscillatory activities at Cz electrodes over the selected time window and frequency band.

The ANOVA for fillers at 130–230 msec interval showed a significant main effect of *Prosody-type*, [*F*(3,42) = 9.83, *p*<.001, *η* ^2^ = .41], and interactions of *Prosody-type*×*Sagittality* [*F*(6,84) = 6.03, *p*<.01, *η* ^2^ = .31], and *Laterality*×*Prosody-type* [*F*(6,84) = 8.37, *p*<.001, *η* ^2^ = .37]. Further simple tests showed that in both task conditions “AN” elicited more negative going deflection in comparison with other kinds of prosodies over frontal-central regions at right hemisphere and midline (*ps*<.01), whereas “AAL” elicited no more negative going ERPs than the “AA” nor “NN” prosodies did over all regions (*ps*>.1, see Figure 5). The ANOVA over 250–450 msec interval revealed a significant main effect of *Prosody-type*, [*F*(3,42) = 7.55, *p*<.001, *η* ^2^ = .35], and two way interactions of *Prosody-type*×*Sagittality* [*F*(6,84) = 6.90, *p*<.001, *η* ^2^ = .33], *Laterality*×*Prosody-type* [*F*(6,84) = 9.36, *p*<.001, *η* ^2^ = .40, and *Prosody-type*×*Task* [*F*(6,84) = 3.18, *p*<.05, *η* ^2^ = .19]. Moreover, the three way interaction of *Laterality*×*Task*×*Prosody-type* was also significant [*F*(6,84) = 4.64, *p*<.01, *η* ^2^ = .25]. The following simple analysis showed that “AN” prosodies elicited more positive going deflection in comparison with “AA” and “AAL” prosodies over both hemispheres and midline electrodes only under emotion task condition (*p*<.05 or p<.01). The analysis of ERSP over 100–600 msec showed similar effect (see Figure 5). The ANOVA showed a significant main effect of *Prosody-type*, [*F*(3,42) = 10.54, *p*<.001, *η* ^2^ = .418], and three way interaction of *Laterality*×*Task*×*Prosody-type* [*F*(6,84) = 4.64, *p*<.01, *η* ^2^ = .25]. The following simple analysis found that the theta band power for “AN” was larger than that for “AA” and “AAL” (*p*<.05) over midline electrodes under emotion task while no difference were found between “AA” and “AAL” prosodies (*p*>.1).

Decreasing mean sound intensity of angry prosodies increased task difficulty, as indexed by the significantly higher error rate and longer reaction time for “NAL” and “AAL” prosodies than for “NA” and other matching prosodies under sound intensity judgment condition. Nevertheless, the modification of mean sound intensity seem to have little effect in the emotional task, as no significant differences in reaction time were observed when participants were instructed to judge emotional congruency for the critical prosodies. In addition, higher error rates and longer reaction times were observed for AAL prosodies under emotion task condition, implying a significant impact on vocal emotion judgment. Since almost 90% of such prosodies were judged as having no emotion deviation, we speculated that the impact of intensity modification might not be categorical. These results were consistent with the finding that reducing the mean sound intensity of angry prosodies would lessen their anger level, but still sound angry in Experiment 1.

As expected, mismatching emotional prosodies (“NA”, “NAL” and “AN”) elicited N2/P3 complex enhancement in comparison with matching prosodies regardless of prosody types and task demands. This result was consistent with the previous finding that deviations in emotional prosodies evoked N2/P3 complex [23]. Given that N2 is frequently associated with detection of context violation and orienting response that directs one's attention to deviation [38], [39] and P3 is related to response decisional processes and context updating [38]–[40], our observation of N2/P3 complex suggested that participants detected the emotion deviation by increasing attention allocation and recruiting greater cognitive resources to integrate the deviation with the preceding context. Moreover, the mismatching prosodies induced enhanced power theta band compared to matching prosodies, consistent with previous finding that expectation violation is associated with theta band power increase [26]–[30]. However, the fillers with only sound intensity modification (“AAL” prosodies) evoked no significant N2/P3 enhancement or theta power increase, suggesting that only modification of sound intensity cannot lead to significant emotional variation.

Central to the present study, we observed that the N2/P3 complex showed prolonged peak latencies and decreased amplitude for “NAL” prosodies than “NA” prosodies regardless of task demands. Furthermore, although both “NA” and “NAL” prosodies induced enhanced theta power relative to matching prosodies in both task conditions, the latter evoked smaller synchronization. As stated by previous studies, the peak latency and amplitude of P3 is a reflection of the degree of match between the stimulus presented and the internal representation of the stimulus relevant for the task [41] while theta power are sensitive to expectation violation salience [33], [34]. The current pattern of electrophysiological data would therefore point to the fact that reducing sound intensity lead to smaller deviation salience. Taken together, these data suggested that sound intensity modification quantitatively affect the emotionality in vocal emotion although it cannot qualitatively change their emotion category.

## Discussion

The present study aimed to address the role of sound intensity and its neural correlates in vocal emotion perception. By explicitly evaluating the anger level of the angry and neutral prosodies before and after their mean sound intensity was manipulated, we found that simply raising the sound intensity of neutral prosody could not make it sound angry while decreasing the sound intensity made angry prosody sound less angry. Moreover, the change of sound intensity of angry prosodies has significant influence on intensity consistency judgment but has little impact on emotional consistency judgment. In concert with these behavioral responses, mismatching prosodies induced typical N2/P3 complex and theta band synchronization regardless of sound intensity level and task requirements. However, mismatching prosodies with reduced sound intensity elicited longer peak latency and smaller amplitude in N2/P3 complex and smaller theta power enhancement. The significance of these findings will be addressed in the following discussion.

By manipulating the mean sound intensity of neutral and angry prosodies, the present study indicated that raising or reducing mean sound intensity could not qualitatively change the anger level of the prosodies. This finding is consistent with previous studies which indicated that it is a specific acoustic pattern, but not a single acoustic parameter alone, that convey vocal emotion [1], [43], and no parameter alone is able to carry the whole emotion information and the intensity contours do not bring any significant information when used alone [16]. However, reducing mean sound intensity decreased the anger level of angry prosodies quantitatively, suggesting that sound intensity do play a role in vocal emotion expressing, in line with the numerous studies which stated that loudness is one of the acoustic cues to convey emotion [1], [42]–[43].

More importantly, the electrophysiological data showed the same pattern of effect. The present study employed cross-splicing method [22] to create deviation in sentence emotional prosody, specifically, the part before the splicing point established the context and generated expectation for the upcoming stimuli, while the part after the splicing point violated the already established context and prediction. Similar to context violation established by oddball paradigm [18], the context violation in emotional prosody was reported to evoke enhanced N2 and P3 in comparison with emotional prosody with no context violation [20], [23]. Consistent with these studies, the deviation in the present study, regardless of intensity modification and task requirement, elicited enhanced N2/P3 complex, indicating that the deviation irrespective of sound intensity modification could be detected and integrated with the preceding context. Besides the N2/P3 complex in time domain, the deviation in both types of mismatching emotional prosodies induced theta band power increase, consistent with the previous studies [31]–[36]. Therefore, it is reasonable to speculate that decreasing the mean sound intensity cannot change the pattern of the emotional deviation. However, despite the similar pattern of results for both “NAL” and “NA” prosodies in time and frequency domain, the data distinct from each other quantitatively, that is, compared with “NA” prosodies, the “NAL” prosodies induced N2/P3 complex with longer peak latency and smaller amplitude as well as smaller theta band power enhancement. Since previous studies suggested that the peak latency and amplitude of N2\P3 complex [23], [43], and theta band power are sensitive to deviation salience [33], [34], the quantitative difference in electrophysiological data seemed to indicate that sound intensity modification in “NAL” prosodies decreased the salience of the expectation violation. Furthermore, the electrophysiological data in both time and frequency domain seemed not to be modulated by task demands. This may suggest that emotional feature has precedence over single acoustic parameter in vocal emotion perception, that is, deviation in emotional feature can surpass the consistency of sound intensity even when the task was to decide sound intensity consistency.

Taken together, the current data demonstrated that sound intensity alone cannot categorically change the emotionality embedded in vocal expression, however, as an integrate part of the acoustic pattern conveying emotion, it can contribute to the emotional significance quantitatively. The question then arises as to why sound intensity has such an effect in vocal emotion perception. Several reasons may account for this. Firstly, if we take emotion as a dimensional construct with several dimensions like valence, activation, potency and intensity [44], sound intensity is a very important cue for intensity or activation dimension [1], [9], [14], [45]. However, if vocal emotion is regarded as several basic emotions, sound intensity may not bring any vital contribution. Secondly, sound intensity is one of the most elementary features of auditory signals, thus it would definitely contribute to any function of auditory signals, including emotion expression, which has been testified by numerous studies [1], [9], [14], [43]. However, because vocal emotion is conveyed by certain combination of acoustic cues, only one parameter alone is unable to carry the whole emotion information [16]. Thirdly, acoustic cues seem to have different status in communicating emotion. Compared with the vital role of pitch [2]–[4], the contribution of sound intensity is relatively minor, as indicated by a recent study demonstrating that while the pitch related parameters assume the most important role in emotion perception (with a ranking score of more than 98%), the role of loudness related parameters is comparably inferior, only with a ranking score of less than 60% [46]. Fourthly, as Juslin and Laukka [42] noted, acoustic cues are used probabilistically and continuously, so that cues are not perfectly reliable but have to be combined. Also, it was suggested that the cues are combined in an additive fashion, and there is a certain amount of “cue trading” in emotion expression. For instance, if one cannot vary pitch to express anger, s/he may compensate by varying loudness a bit more [47].

There are some limitations that should be taken into consideration when making conclusion. Firstly, only angry and neutral prosodies recorded by one speaker were used, which constrained the generalizability of the current conclusion. To validate the current conclusion across various emotions, more emotion types and speakers should be employed in further study. Secondly, since only two levels of sound intensity were employed and the raising pattern of comparison (“NN”, “AN”, and “ANH”) was absent in Experiment 2, a definite conclusion of continuously rather than categorically encoding of sound intensity can not be reached. Thirdly, since only Mandarin materials and Chinese subjects were employed in the current study, given the acoustic parameters to express emotion are likely to vary across languages [48], the reported findings may not be entirely generalizable and cross-culture studies are needed. However, despite these limitations, the present study did provide some evidence, especially electrophysiological evidence for the first time, that sound intensity play a specific role in vocal emotion communication, and suggested that it should be with caution when sound intensity is taken as a control parameter in neurocognitive studies of vocal emotion.

In sum, the present study, in conjunction with previous studies, demonstrated that sound intensity is an important acoustic cue for vocal emotion decoding. Although it could not categorically contribute to emotionality embedded in vocal emotion, sound intensity can quantitatively affect its emotional significance. Hence, sound intensity should not simply be taken as a control parameter and its unique role needs to be specified in vocal emotion studies.

## Materials and Methods

### Experiment 1

#### Participants

Eighteen right-handed native speakers of Mandarin Chinese (nine women, aged 19–25, mean 22.44) were recruited to participate in the experiment. All participants reported normal auditory and normal or corrected-to-normal visual acuity and no neurological, psychiatric, or other medical problems. The study was approved by the Institutional Review Board of Institute of Psychology, Chinese Academy of Sciences, and participants gave written informed consent and received monetary compensation.

#### Stimuli

**Figure 6:**
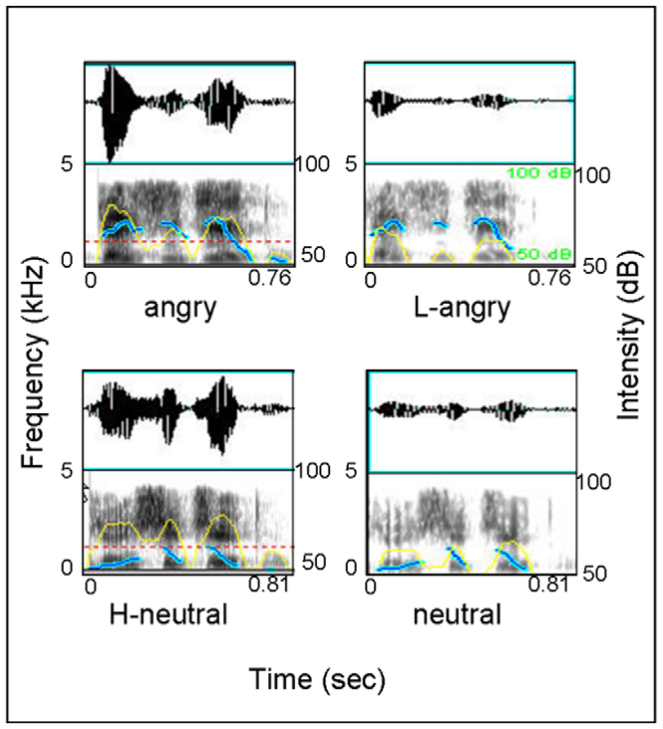
Acoustic feature for four prosody types used in Experiment 1. The dataset consists of oscillogram (up) and voice spectrographs (down) with uncorrected pitch contours (blue line) and intensity contours (yellow line) superimposed. As shown, angry prosodies have higher mean *F*_0_ (197 Hz vs. 132 Hz, *t*(49) = 28.12, *p*<.001) and intensity (70 dB vs. 63 dB, *t*(49) = 23.18, *p*<.001), and faster speech rate (206 ms vs. 216 ms per syllable, *t*(49) = 5.43, *p*<.01) than neutral prosodies, however, the intensity modified H-neutral and L-angry prosodies share same level of intensity with angry and neutral prosodies respectively while other acoustic parameters remain unchanged.

The original materials were 50 phrases of four syllables, which were the last syllables cut from sentences used in a previous study [23]. All these sentences were neutral in content, and recorded in angry and neutral prosodies. The result of acoustic analysis indicated that these prosodies had typical acoustic features reported by Banse and Scherer [1]. The phrases were manipulated using Audition software as following: the mean sound intensity of angry prosody was reduced to the level of neutral prosody to create low-sound -intensity angry (L-angry) prosody, and the mean sound intensity of neutral prosody was raised to the level of angry prosody to create high-sound-intensity neutral (H-neutral) prosody (see Figure 6 for a graphic illustration and acoustic parameters). Two hundred phrases, including four types of emotional prosodies (angry, L-angry, neutral, H-neutral), were presented to participants altogether.

#### Procedure and data analysis

Participants were asked to rate each phase along anger level (4-point Likert scale with 1 being no angry and 4 extremely angry). Sounds were presented pseudo-randomly over four blocks of trials, each of which was comprised of 50 trials that were broadly equivalent in the number of four types of stimuli. Each sound presentation, via headphones, was followed by a visual cue and Likert scale on a computer monitor. Responses on the Likert scale were performed with a computer keyboard with a time limit for 3000 msec. The inter-trial interval was 1500 ms. Practice trails were used to familiarize participants with the procedure and excluded from data analysis. The mean rating value of angry level and reaction times for four types of prosodies was calculated across the 18 subjects first, and then subjected to repeated measures analysis of variance (ANOVA) with *Prosody-type* as within-subject factor.

### Experiment 2

#### Participants

Sixteen right-handed native speakers of Mandarin Chinese (nine women, aged 22–25, mean 23.44) were recruited to participate in the experiment. None of them participated in Experiment 1. All participants reported normal auditory and normal or corrected-to-normal visual acuity and no neurological, psychiatric, or other medical problems. This study was approved by the Institutional Review Board of Institute of Psychology, Chinese Academy of Sciences, and participants gave written informed consent and received monetary compensation. One participant was excluded from analysis because of excessive artifacts during the EEG recording session.

#### Stimuli and experimental procedure

**Figure 7:**
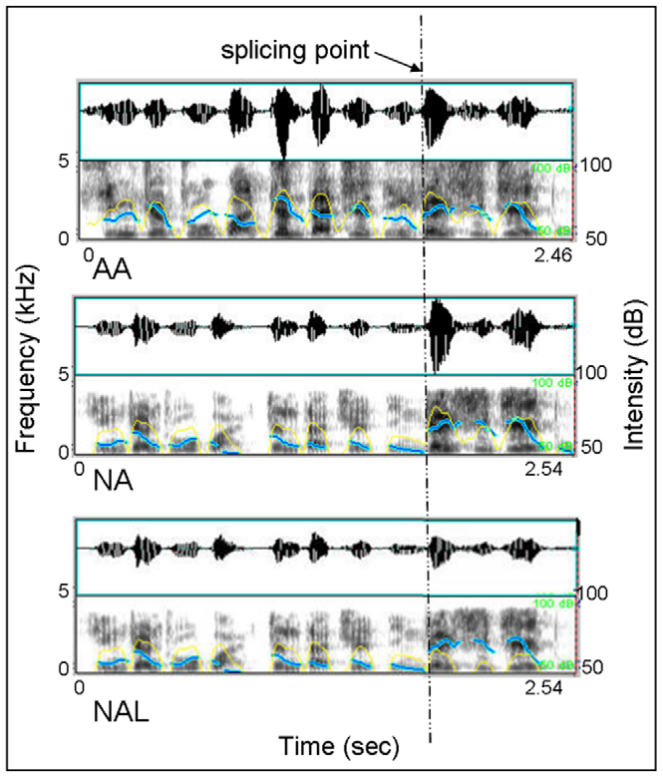
Illustration of the splicing procedure and acoustic feature of three types of prosody used in Experiment 2. As in Experiment 1, the dataset consists of oscillogram (up) and voice spectrographs (down) with uncorrected pitch contours (blue line) and intensity contours (yellow line) superimposed. (Abbr.: AA—all Angry; NA—Neutral-to-Angry; NAL—Neutral-to-low Angry). The correct response was “no-change” for AA and “change” for both NA and NAL under emotion task, whereas under sound intensity task, the correct responses were “no-change” for both AA and NAL but “change” for NA. Moreover, the fillers provided the counterbalance responses under both tasks.

The original stimuli were 50 sentences used in the previous study [23]. All sentences were with neutral semantic content and produced by a trained male actor in neutral and angry prosodies. The original recordings were cross-spliced to get “**n**eutral-to-**a**ngry (NA)” and “angry-to-neutral (AN)” prosodies. Then, the sound intensity of the second part of “**a**ll **a**ngry (AA)” and NA prosodies were reduced to the neutral prosody level to get two other types of prosodies (AAL, NAL, see Figure 7 for a graphic illustration). The mismatching “NA” and “NAL” prosodies together with matching angry “AA” prosodies served as critical materials, while 50 each “all neutral, “NN”, “AN” and “AAL” prosodies served as fillers to confuse subject's prediction of the occurrence of emotional or sound intensity deviation. All together, 300 sentences for each task were presented to participants aurally by headphones.

To familiarize subjects with the feature of sentences and experimental procedure, sample sentences that fit a specific prosodic category were presented to the participants, followed by descriptions of their emotional feature or intensity contour variation for emotion judgment and intensity judgment respectively. And then a practice session with feedback was given. The experimental session was administered to participants only when they achieved a stable 80% correct response rate in the practice session.

In experimental session, sentences were presented in a pseudo-randomized order in six blocks of 50 trials while each participant was seated comfortably at a distance of 115 cm from a computer monitor in a sound-attenuating chamber. In each block, sentences from the same prosodic type were presented in no more than three consecutive trials. Each trial began with a fixation cross in the center of the monitor for 300 ms, and then the sentence was presented while the cross remained on the screen. Participants were instructed to respond as accurately and quickly as possible whether the emotion feature or the intensity contour of the sentence was changed by pressing the “J” or “F” button on the keyboard after they heard the whole sentence. The order of the two tasks and the button for “yes” and “no” were counterbalanced across participants. The inter-trial interval was 2000 ms. Participants were asked to look at the fixation cross and avoid eye movements during sentence presentation.

#### Electrophysiological recording and analysis

EEG was recorded with 64 Ag-AgCl electrodes mounted in an elastic cap (NeuroScan system). EEG data were referenced online to the left mastoid. Vertical electrooculograms (EOGs) were recorded supra- and infra-orbitally at the left eye. Horizontal EOG was recorded from the left versus right orbital rim. EEG and EOG were digitized at 500 Hz with an amplifier bandpass of 0.01–100 Hz including a 50-Hz notch filter and were stored for off-line analysis. Impedances were kept below 5 kΩ. After preprocessed using NeuroScan 4.3 (eye movements and electrode drifting screening) and re-referenced offline to the algebraic average of two mastoids, EEG data were segmented to 3000-msec epochs time-locked to the splicing points of mismatching prosodies and the corresponding points of matching prosodies, starting 1000 msec prior to the onset splicing point (the 3000-msec epoch was used for data analysis, while only 1200-msec epoch with 200-msec baseline was illustrated in the figures).

For ERP analysis, segments were first baseline corrected 200 msec before the start of the sentences and then 200 msec before the splicing point after a low-pass filter of 30 Hz. Trials with artifacts exceeding the amplitude of ±90 µV on any channel and wrong responses were excluded from the averaging and more than 35 trails per condition remained for averaging. ERP waveforms were computed separately for three types of prosodies in both task conditions. The extracted average waveforms for each participant and condition were used to calculate grand-average waveforms and subjected to repeated measures ANOVAs. The grand average ERPs for each condition are illustrated in Figure 3. Visual inspection of the waveforms revealed that all types of prosodies elicited a typical positive-negative-positive sequence of ERP components. While the first positivities elicited by the three types of prosodies were hardly distinguished from each other, the ERPs for mismatching (“NA” and “NAL”) and matching (“AA”) prosodies started to differentiate at about 130 msec across task requirement. These differences were manifested by a centrally peaking N2 during 130–230 msec and a centrally peaking but broadly distributed P3 at 250–450 msec intervals in the mismatching-matching difference waves. Mean voltage for each condition at 130–230 and 250–450 msec intervals were averaged for the left frontal (F7, F5, F3, FT7, FC5, FC3), middle frontal (F1/2, FZ, FCZ, FC1/2), right frontal (F8, F6, F4, FT8, FC6, FC4), left central (T7, C5, C3, TP7, CP5, CP3), middle central (C1/2, CZ, CP1/2, CPZ), right central (T8, C6, C4, TP8, CP6, CP4), left posterior (P7, P5, P3, PO7, PO3, O1), middle posterior (P1/2, PZ, POZ, OZ), and right posterior (P8, P6, P4, PO8, PO4, O2) regions (for regional averaging, see [49]). Repeated measures ANOVAs were conducted to test these effects with *Task* (emotion judgment vs. intensity judgment), *Prosody-type* (“AA”, “NA” and “NAL”), *Laterality* (left vs. midline vs. right), *Sagittality* (frontal vs. central vs. posterior region) as within-subject factors. Moreover, to further clarify the effect of sound intensity modification, we conducted an repeated measures ANOVA on peak latencies and amplitudes (baseline to peak) of the N2 and P3 components at corresponding intervals of the difference ERPs (“NA-AA” *versus* “NAL-AA”) with *Electrode* (F3, FZ, F4, FC3, FCZ, FC4, C3, CZ, CZ, C4, CP3, CPZ, CP4, P3, PZ, P4, PO3, POZ, PO4), *Task*, and *Prosody-type* as within-subject factors. In addition, Although the “NN”, “AN” and “AAL” prosodies were mainly included as fillers, to verify the effects of emotion change in “AN” prosodies and sound intensity change in “AAL” prosodies, the brain responses for all these fillers were analyzed including “AA” prosodies as baseline in a separated repeated measures ANOVA with the same within-subject factors as those for critical prosodies. The degrees of freedom of the F-ratio were corrected according to the Greenhouse–Geisser method in all these analyses.

For event related oscillation (ERO) analysis, induced spectral EEG activity was assessed by creating event related spectral perturbations (ERSP) using a complex sinusoidal wavelet transform procedure as implemented in EEGLAB [50]. The resulting complex signal provides an estimate of instantaneous power for each time point at frequencies of 3–100 Hz. This procedure is done on each trial, and then power values are averaged across trials. Power values were normalized with respect to a −200 to 0- msec prestimulus baseline and transformed into decibel scale (10*log10 of the signal). We used an EEG epoch window of −1000 to 2000 msec from each event to ensure that edge effects would not contaminate our windows of interest, and visual inspection confirmed that edge effects did not extend into our time windows of analyses. The mass-univariate approach implemented in the statcond function of EEGLAB toolbox was used to find out the frequency band and time window that the ERSP values significantly distinguished. And then, based on the mass-univariate analysis, for each subject, the net ERSP values within theta band (4–6 Hz) during time points of interest (200–600 msec) for each prosody type under each condition were averaged for nine regions with same electrodes and participanted to repeated measures ANOVAs with same factors used in ERP analysis. Only the data at Cz was illustrated as the topographic distributions of power exhibited a fronto-central peak that was maximal around Cz in all conditions (see Figure 4).
